# Examining the Relationship Between Urinary Incontinence and Women’s Physical Activity Engagement: Barriers and Disclosure Patterns

**DOI:** 10.3390/healthcare13080856

**Published:** 2025-04-09

**Authors:** Amanda J. M. Gard, David Lavallee

**Affiliations:** Department of Health, Sport and Wellbeing, Abertay University, Dundee DD1 1HG, UK; a.gard1300@abertay.ac.uk

**Keywords:** physical activity, urinary incontinence, women, health

## Abstract

Background: This study investigates the relationship between urinary incontinence (UI) and women’s participation in physical activity (PA). Women are less active than men across their lifespan, and while interventions aim to bridge this gap, the unique challenge posed by UI remains underexplored. UI disproportionately affects women and often results in reduced self-confidence and avoidance of PA. Methods: Employing a mixed-methods design, the study utilised an online survey (n = 345) and semi-structured interviews (n = 14) to explore women’s experiences of UI during PA and its perceived impact relative to other barriers. Results: Findings reveal that UI ranks prominently among barriers to PA, yet disclosure is infrequent without direct prompting. Participants highlighted anxiety, embarrassment, and the inadequacy of PA environments in accommodating UI-related needs as critical deterrents. Interviews further uncovered a lack of practitioner knowledge regarding UI, with many women favouring privacy-centric approaches to address their concerns. Conclusions: The study underscores the necessity for tailored interventions, practitioner education, and inclusive PA environments to enhance participation and mitigate UI’s impact. These findings contribute to broader efforts to promote gender equity in PA and improve women’s health outcomes.

## 1. Introduction

Understanding the barriers women face in their PA participation is important if interventions are to successfully tackle the lower levels of PA that women experience in comparison to men. The gap in PA levels is well discussed in the literature, with women experiencing lower levels of PA than men across their entire lifespan [1,2]. However, despite a plethora of interventions aimed specifically at increasing women’s PA levels, the gap between men and women has changed little over time, as can be observed in the sex-stratified data of national health reports [3]. Improving women’s PA levels is especially important to their health, particularly as they age, since women live longer than men but spend more of their longer lives in poorer health and frailty [4]. Frailty is more prevalent in older women, and PA has been shown to effectively reduce its progression. This helps women maintain mobility for longer and decrease the risk of disability often linked to older age [5,6]. Physical activity has also been shown to be effective in managing or preventing many of the diseases common to women, including cardiovascular disease, type 2 diabetes, and various cancers [7,8]. It also supports mental health by reducing the risk of depression and cognitive decline, such as Alzheimer’s [9,10,11,12]. It is, therefore, crucial that women are encouraged and supported in improving their PA behaviours.

The first step in designing effective PA interventions that help increase women’s PA rates and help maximise the potential health benefits for this demographic is to understand the barriers that women experience to their PA participation. There is a large and growing body of evidence exploring women’s PA barriers, with the most reported, including low motivation, a lack of time, and fatigue [13]. In addition to these well-documented barriers, women experience unique physiological challenges, such as those related to pregnancy, menstruation, and menopause, which can further impact their participation in PA [14,15]. While the barriers to PA faced by women are complex, one notable issue that has received insufficient attention in both academic research and practical interventions is urinary incontinence (UI). UI is a condition defined as any involuntary loss of urine from the bladder [16], and it disproportionately affects women [17], with prevalence rates estimated to range between 5% and 70% depending on the population and diagnostic criteria used, though most studies report a rate in the 25–45% range [18]. In the literature, UI has been identified as a significant barrier to PA for many women, particularly in activities that involve high-impact movements or strenuous effort, such as running, jumping, or weightlifting [19]. The fear of leakage during exercise can cause embarrassment, anxiety, and reduced self-confidence, ultimately leading to the avoidance of PA altogether [20,21]. This is particularly concerning given the cyclical relationship between UI and physical inactivity; while PA can help prevent and manage UI through pelvic floor muscle strengthening and weight-loss management, physical inactivity can also exacerbate the condition [22].

Despite its relevance, UI is often overlooked in public health discourse on PA. Existing research appears to focus on broader barriers such as time constraints and motivation, neglecting to explore how health-related conditions such as UI influence women’s PA behaviour. Furthermore, many women may not disclose their experiences with UI, even in clinical or research settings, due to feelings of embarrassment or normalisation of the condition as an inevitable consequence of ageing or childbirth [23]. This lack of disclosure creates a significant gap in understanding the true impact of UI on women’s lives and their engagement with PA. The reluctance to disclose UI is compounded by societal stigma and the perception that it is a private issue. Research indicates that many women feel isolated in their experiences, believing that they are alone in dealing with UI, despite its prevalence [24]. This lack of open discussion perpetuates a cycle of silence, where women’s needs are not addressed in healthcare or PA-related interventions. Disclosure is further hindered by a lack of awareness about treatment options; many women are unaware that UI is a treatable condition and may not seek help from healthcare professionals [25].

The barriers to disclosing UI are particularly pronounced in the context of PA. Women may feel that discussing their condition with fitness instructors, coaches, or peers would expose them to judgement or misunderstanding [20,26]. Additionally, the design of many exercise environments, including the types of activities offered and the facilities provided, often fails to accommodate women with UI. For example, the lack of private changing rooms or accessible toilets can deter women from participating in PA [21].

While there is growing recognition of the need to address gender-specific barriers to PA, research on the impact of UI on women’s PA participation remains limited. Most existing studies focus on clinical interventions for managing UI and scarcely explore the wider psychosocial implications of the condition. There is, however, a particular lack of qualitative research exploring women’s lived experiences of UI in the context of PA. Such research is essential for understanding the complex ways in which UI affects women’s decision-making and behaviour, as well as for identifying strategies to support women’s participation in PA and overcome barriers. It is also unknown to what extent women disclose UI in the PA environment, nor whether women would voluntarily mention UI as a barrier in unprompted discussions or whether they require prompting to elicit this information. This gap highlights the need for research that explores the dynamics of UI disclosure and the factors that influence women’s willingness to discuss UI in the PA environment. Given the identified gaps in the literature, this research seeks to contribute to a more comprehensive understanding of the barriers women face to their PA activity participation and to inform the development of targeted interventions that promote inclusivity and support for women with UI. Specifically, the study aims to:

1. Aid practitioners’ knowledge of urinary incontinence’s impact on women’s PA participation through the exploration of women’s perceptions and experiences of urine leakage during exercise;
2. Test whether women will disclose their UI status unprompted when questioned about barriers to PA;
3. Gain an understanding of where UI ranks amongst the most common barriers women experience to their PA participation;
4. Gather insights into women’s UI disclosure both in and out of the PA environment.

## 2. Materials and Methods

### 2.1. Study Design

**Figure 1:**
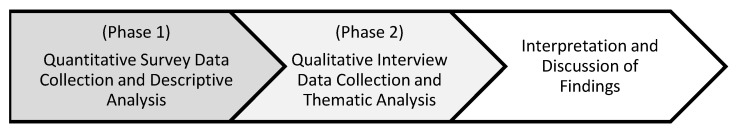
Explanatory sequential mixed methods research design.

To meet the aims of this study, a mixed methods research design was undertaken using an explanatory sequential strategy (Figure 1), which involves collecting quantitative data before analysing qualitative data to help explain results [27]. It is due to this that the method was primarily chosen, as it allows the qualitative data gathered in the second phase to help explain the quantitative findings from the first phase, providing an in-depth and rich body of evidence.

The first phase of the study was designed to meet study aims 1–3 and employed a research design that collected quantitative data through an online survey to examine whether women would disclose their UI status or report the condition as a barrier to PA without prompting. The survey also sought to understand where UI is ranked as a barrier to PA relative to more commonly reported barriers to PA and to provide a greater understanding of women’s experiences and perceptions of leaking urine during PA participation. The second phase was primarily focused on study aim 4 and collected qualitative data through individual semi-structured interviews to explore UI disclosure and perceptions of UI in the PA environment in greater depth and to help explain the quantitative findings.

### 2.2. Procedure

#### 2.2.1. Phase 1 Survey Participants and Recruitment

Due to the purpose of the survey research, a multi-stage sampling strategy was undertaken to recruit participants. Firstly, convenience sampling was used, as the researcher was an (inactive) member of a large private women’s group on social media, which provided convenient access to many potential participants. Recruiting within a closed group for the first time also helped limit the chance of recruiting women who had participated in the researcher’s previous studies, which had been advertised via the researcher’s personal social media profiles. This was important, as it was thought previous study participants were likely to be more aware of the researcher’s area of study and thus UI could already be in participants’ thoughts as they completed the survey, resulting in biased question responses. By targeting new participants and framing the test survey around common barriers to PA rather than UI as a barrier specifically, the risk of biased responses could be minimised. Additionally, the invitation to participate in the survey was posted to the chosen social group specifically, as the group members reflected a diverse range of ages, backgrounds, education levels and life stages. It was hoped such considerations would help maximise the gathering of a wide range of views and experiences from women who exercise with UI or cease exercising because of UI symptoms and provide insight into how UI manifests as a barrier to PA participation, thereby producing an in-depth body of evidence to improve physical activity and health (PAH) practitioners’ and policymakers’ knowledge of UI and its impact on PA participation.

Criterion sampling was applied to confirm participant demographics met the requirements of the study. The first study requirement was that only female participants were recruited due to stress urinary incontinence being the most common form of UI affecting women in the PA environment, as reported in the literature [28,29]. To meet this criterion, participant responses confirmed their sex. In addition to being female, participants were required to be at least 18 years old. No maximum age for participants was set, as little appeared known of whether age or stage of life influences women’s perceptions or experiences of UI. Having no upper age limit may also provide an opportunity to gain additional insight into whether age or life stage affects the willingness of women to voluntarily disclose their UI status.

Certain responses ended participation immediately to focus on the target group. Due to the recruitment materials avoiding direct mention of UI, the initial pool included women both with and without UI. One question, halfway through the survey, asked participants the following question: “As an adult, have you ever leaked urine unexpectedly, or felt you might leak urine unexpectedly, during physical activity?” Only those reporting such experiences were prompted about UI as a barrier to PA (i.e., “Is leaking urine a barrier to your physical activity participation or does it make you alter your participation in any way?”). Then, only those answering that UI is a barrier to their PA were able to continue with the survey and explore UI in relation to other barriers to PA. Participants who indicated no UI experiences or barriers exited the survey at this point and were thanked for their participation.

In total, 345 participants with and without UI began the survey, and 189 women indicated that they experienced UI during PA. Only those participants who completed the entire survey were invited to consider participating in phase two, after which the survey concluded with a thank you for participating. At each stage where participants were removed from the survey, a link was provided to the UI information pages of the National Health Service (NHS) in the hope of encouraging participants to seek help for their UI. The general demographics of survey participants’ can be viewed in Table 1.

#### 2.2.2. Phase 2 Interview Participants and Recruitment

Criterion sampling was used to recruit participants to the interview stage of the study; participants were required to have fully completed the phase 1 survey and have provided their email addresses, as well as given consent to be contacted regarding phase 2. Of the participants who completed the survey, 109 participants provided their email addresses and agreed to take part in the interview stage. The study aimed to recruit 12 women to interview, and to ensure a broad range of experiences would be represented, the different responses participants gave to the survey were considered, and participants were grouped according to their survey question responses, specifically. From these groupings, participants were randomly drawn, ensuring at least one participant from each group, culminating in a shortlist of 12 women who were then invited for interviews. Participants were asked to respond to the invitation within 7 days, and any participants who declined the invitation or who did not respond within the time frame were replaced with a participant from a similar group. This process continued until 12 participants confirmed their willingness to be interviewed. Due to two interviews yielding little information of note, an additional two participants were interviewed, bringing the total number of women participating in the phase two interviews to 14 (lasting between 28 and 68 min). Participant demographics can be viewed in Table 2.

All interviewees were emailed a Participant Information and Consent Form and asked to electronically sign the consent section if they were happy to continue. Participants were also asked to provide their availability, which was then used to schedule interviews. All interviews were scheduled to be held online via Microsoft Teams and participants were requested to inform the researcher if they were uncomfortable with being interviewed in this manner. No participant raised any concerns. Participants were also asked to confirm their location; this was deemed helpful as, due to differing healthcare systems and opportunities available for UI treatment globally, it was thought important to ensure all participants were UK residents for a better interpretation of the research findings. Interviews were scheduled at the participants’ convenience, and all 14 took place within a 10-day period.

### 2.3. Data Collection

#### 2.3.1. Survey Materials and Data Collection (Phase 1)

Once recruited, participants completed an online survey consisting of 15 closed-ended questions, including 5 general demographic questions and 10 questions which aimed at testing whether women with UI would disclose their UI status unprompted and to identify where UI is ranked in relation to other, more commonly reported PA barriers. An online survey was the chosen method of data collection due to the ease with which suitable participants across a large geographical area can be reached at little administrative cost [30]. Administering an online survey for this study was also fortuitous as it allowed data collection to continue, albeit at a slower pace, during a period of COVID-19 restrictions. Compared to open-ended questions, closed-ended questions have the potential for eliciting quicker responses from participants, which can lead to an increased response rate [31].

The survey questions were constructed in line with the aims of the study and the literature and with consideration to the recommendations made by Storey and Tait [31]. For example, the language was succinct and simple to maximise easy understanding, and the survey took less than 10 min to complete. Prior to distribution, five female acquaintances who had taken part in previous studies and who lived locally to the researcher, were asked to complete a printed copy of the survey to gain feedback and test the question structure, language, and understandability. Obtaining feedback and testing the survey is important, as doing so can increase completion rates and improve survey effectiveness, whilst also identifying any issues prior to data collection commencing [31]. Initially, the question designed to ascertain the participant’s sex was reported as difficult to understand and was reworded several times until it was understandable and adequately identified only females. Additionally, some questions and instructional paragraphs were identified as ‘too complicated’ and were simplified before being re-tested and the survey opened to participants. The minimum sample size for phase 1 was calculated at 340 participants; after 4 weeks, the survey was closed with a total of 345 participants.

#### 2.3.2. Interview Schedule and Data Collection (Phase 2)

Prior to the interview start, participants were verbally informed of their right to withdraw from the study at any point [32] and were notified that all reasonable steps to hide their identity would be taken in all documentation and output data. It was acknowledged to each participant that UI may be a sensitive topic to discuss, and as such, if the participant felt uncomfortable at any time, the researcher would be happy to take a break or cease the interview at the participant’s behest without any form of justification being required. Where the research topic may be viewed as sensitive, the literature suggests researchers should develop a rapport with participants prior to the interview, which will help interviewees relax and feel safe in discussing their condition and experiences in-depth and honestly [33,34]. The literature suggests rapport building is more effective when interviews occur in person, face-to-face, and before the interview takes place [33,35]; however, recent evidence suggests establishing rapport can be as effective in online interviews, with participants equally likely to provide in-depth disclosure [36,37]. Rapport building began with the invitation to take part in phase two by the researcher providing as much detail on the study/interview aims and participant requirements as possible, as recommended by McGrath et al. [33]. Elmir et al. [38] and Dickson-Swift et al. [39] also suggest researchers self-disclose their own honest experiences of the topic being discussed in the interview, particularly if it is a sensitive topic, as this can build rapport and trust which may encourage participants to discuss their own experiences more openly. This has been a successful strategy for the researcher in previous interviews and was therefore similarly employed for the current study.

An interview schedule was used to guide the discussion using open-ended, semi-structured questions which allow participants the opportunity to express in their own words their perceptions/feelings towards UI and how it affects their PA participation whilst also allowing the researcher the flexibility to explore participant responses with follow-up questions [32]. The questions in the schedule were influenced by the survey findings, and the schedule also served as a valuable tool to ensure interviews remained on topic [40].

Once the researchers were certain a rich dataset was secured, participants were given an opportunity to think about the interview and the points of their disclosure, as well as to add any additional information they thought important. It was also reconfirmed by the researcher (first author) that once the audio from their interview had been transcribed, the video would be deleted to ensure their anonymity. Transcription was undertaken by the researcher, aided by the online transcription software ‘Otter.ai’ (https://otter.ai/) immediately after each interview. Transcribing quickly allowed the researcher to make any notes regarding context or speech patterns that may be useful in explaining the points being discussed. Once transcription was completed, each participant was emailed and given 21 days to request to view their transcript to ensure they were happy for quotes to be published, particularly as some interviewees had disclosed personal details about their health and feelings. No requests for this were received by the researcher, and after 21 days, all transcripts were pseudonymised by allocating a unique participant code to each. On the few occasions where participants had referred to others or places by name in their interview, the names used were also changed to help maintain anonymity, and the original transcript was overwritten. Only at this point were the video recordings deleted, and data analysis of the interview transcripts began.

### 2.4. Data Analysis

#### 2.4.1. Phase 1 Survey Data Analysis

The survey data were gathered to test whether women who experience UI during PA would disclose UI as a barrier to PA without being prompted and to provide insight into the ranking of UI as a barrier to PA compared to other barriers reported. Survey Hero (www.surveyhero.com, accessed on 3 April 2025) was used to design the survey and produce analytics in line with the study aims. Analysing survey data can take some time depending on the design complexity and response rate, but it can also be used to help identify potential participants for further study [31]. In this case, the survey identified suitable participants for phase two of the study.

#### 2.4.2. Phase 2 Interview Data Analysis

The data gathered from the interview phase was first transcribed verbatim immediately post-interview. Thematic analysis was used to identify the patterns and themes within the data [32,41]. The flexibility of thematic analysis is useful when the topic area is little researched, and because it can help identify patterns within the data, it is well suited to research that explores individuals’ feelings and perceptions [42].

Braun and Clarke’s [41] six-phase thematic analysis framework was used inductively to guide the thematic analysis alongside the step-by-step guide from Maguire and Delahunt [42].

**Figure 2:**
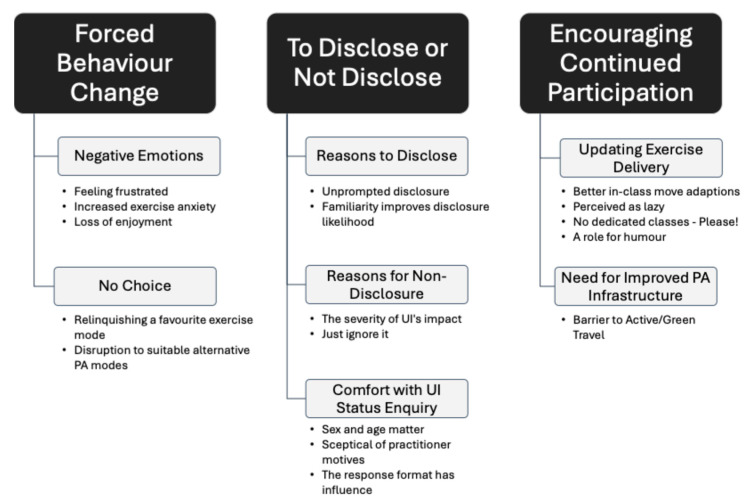
Thematic map.

Using Quirkos 2 software (https://www.quirkos.com/), the transcripts were first read and re-read before initial codes were manually highlighted. Quirkos 2 was chosen to aid analysis as it allowed the researcher to maintain control of the analysis process whilst providing a visual illustration of the identified themes. It also allowed transcripts and recorded participant demographics to be easily curated and compared. Initial codes were colour-coded and sorted into broad themes, at which point the codes and themes were reviewed to ensure a coherent fit with the data and research question. Themes were then defined, and a thematic map was produced (Figure 2). The thematic analysis resulted in 3 general dimensions, 7 themes and 17 sub-themes, with 33 meaningful units being identified in total, and forming the basis of this study. The 3 general dimensions identified were ‘Forced Behaviour Change’, ‘To Disclose or Not Disclose’, and ‘Encouraging Continued Participation’.

## 3. Results and Discussion

### 3.1. Phase 1 Survey Findings

#### 3.1.1. UI Prevalence Rate

Whilst the study concentrated primarily on women who leak urine during PA, the survey began by also including responses from women who do not experience UI. Of the 345 women who responded to the survey, 65.8% reported leaking during PA participation.

Whilst there has been an increase in research in this area recently, comparative evidence for UI prevalence during PA is difficult to locate in the literature, as most studies include only current exercisers, often athletes participating in sports, and rarely include leisure-time PA participation or those who have ceased exercising due to UI symptoms, or those who have never exercised. For example, Nygaard and Shaw [43] found studies included in their review reported a prevalence rate of UI in women participating in sports of between 14% and 80%, with higher intensity sports such as trampolining eliciting higher UI prevalence rates than those of lower intensity. Teixeira et al. [44] reported in their review of female sports athletes an overall prevalence rate of 36% across all types of sports. Neither study specified that leaking during leisure-time or domestic-related PA was included, nor did they include women in their prevalence figure who had ceased sport participation entirely due to UI symptoms. However, the literature search did yield a more recent study whose participant demographics and definition of PA were similar to those of the current study, which reported 60.8% of their female participants leaked urine or were concerned they might leak urine during exercise [45]. This figure is relatively similar to the current study’s findings but is considerably higher than the rate of UI reported in sportswomen. This may be due to the older average ages of the women in both these studies compared to that of women athletes in many sports studies. Both the quoted study by Dakic et al. [45] and the current study also captured responses from women who had stopped being physically active due to UI symptoms, which are often omitted from sport/athlete UI studies. By including women in midlife and older, those who have ceased PA due to symptoms, and PA derived from work and domestic tasks, a more accurate prevalence rate is deduced.

#### 3.1.2. Disclosing UI in the PA Setting

Given the prevalence of UI during PA reported by participants in this study, knowing where it ranks in comparison to other commonly reported barriers seems important. However, the literature suggests women are reluctant to self-disclose symptoms of UI, with studies finding only an estimated 30% to 34% of women will raise the condition with a medical practitioner. Older women, who are the demographic at greatest risk of experiencing UI, are also the least likely to self-disclose to clinicians [46]. Disclosure of symptoms in the clinical setting appears to be influenced by multiple factors, including anticipated clinician reactions, stigma, and embarrassment [47]. Even when the opportunity is presented, or when symptoms severely impact quality of life (QoL), UI symptoms often remain unreported [48]. It is unknown whether these factors also influence women’s likelihood of self-disclosing UI symptoms in the PA environment, resulting in PA practitioners’ unawareness of UI’s impact and missing the opportunity to address women’s PA participation more effectively. Therefore, the current study sought to test whether women would report UI as a barrier to PA without prompting and to establish its ranking in relation to other barriers.

**Figure 3:**
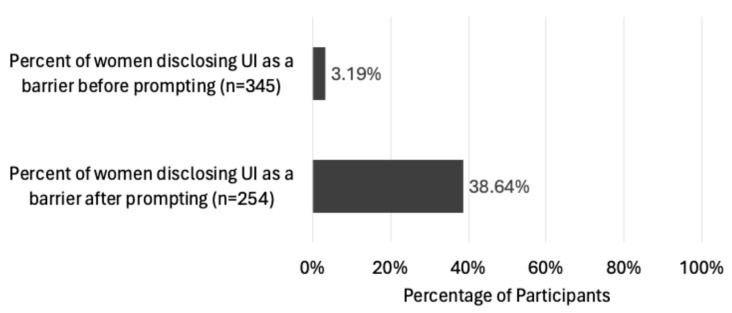
Percentage of women disclosing UI unprompted/prompted.

When, participants were asked to list all barriers affecting their PA participation, without directly referencing UI, 3.2% of 345 participants mentioned UI, leaking urine, or toileting (Figure 3). This figure is suggestive of the rates of unsolicited UI symptom disclosure in exercise/sports settings where athletes rarely report the condition to their coaches [26]. The reasons for this, as noted in studies, appear similar to those cited for non-disclosure to medical professionals, such as embarrassment, stigma, and a perceived lack of available treatment [47].

In addition to the unprompted findings, Figure 3 also illustrates that when the same participants were specifically asked whether UI was a barrier, 38.6% confirmed it was. This suggests that women in the PA environment are unlikely to divulge UI as a barrier without being specifically asked. This finding may indicate why UI remains unmentioned in much of the literature regarding women’s barriers to PA. However, to be more confident that the omission of UI as a target of interest in intervention studies is due to women under-reporting UI and PAH practitioners thus being unaware of its significance as a barrier, rather than UI not actually being perceived as impacting participants’ PA, participants were also asked to rank UI against the barriers they had reported in the survey. Requesting participants to rank UI against other barriers they experience allows for a better understanding of whether UI does indeed impact PA behaviour. It also provides some indication as to the severity of UI’s impact on women’s PA participation.

#### 3.1.3. Ranking UI as a Barrier to PA

There is a large body of literature investigating the common PA barriers women have reported experiencing, which include a lack of time, family responsibilities, additional health concerns, lack of a workout partner, and a lack of motivation [49,50,51]. While the general barriers listed by participants were not analysed as part of this study, there appeared to be no surprising submissions compared to those listed in the literature.

**Figure 4:**
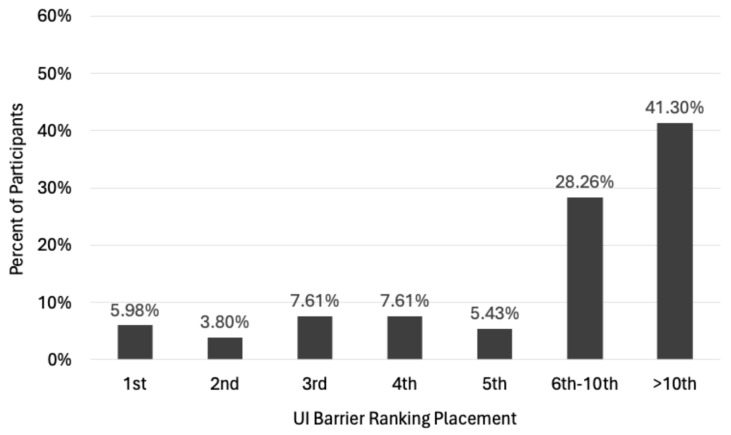
Ranking of UI in relation to participants’ top 10 PA barriers (n = 184).

When asked where UI ranks as a barrier amongst the other barriers experienced, 58.7% in total reported UI as a top 10 barrier, 30.4% of those placed UI in their top 5 barriers, and 9.8% reported UI as their first or second highest ranked barrier (Figure 4).

The findings indicate that over 30% of participants with UI during PA perceive the condition as a top 5 barrier to their participation and suggest that interventions which aim to support women in managing UI in the PA environment should be included to help combat the drop in PA levels experienced, particularly as women age. Given that previous studies have shown exercising women to be at a lower risk of developing all forms of UI [52], and some forms of exercise have been proven to be effective at treating UI symptoms [53,54], the PA environment may also serve as a suitable UI screening and primary treatment. However, PA practitioners should be prepared to ask women about their UI status rather than expecting women to raise it as an issue voluntarily.

#### 3.1.4. Effect of UI on PA Participation

**Figure 5:**
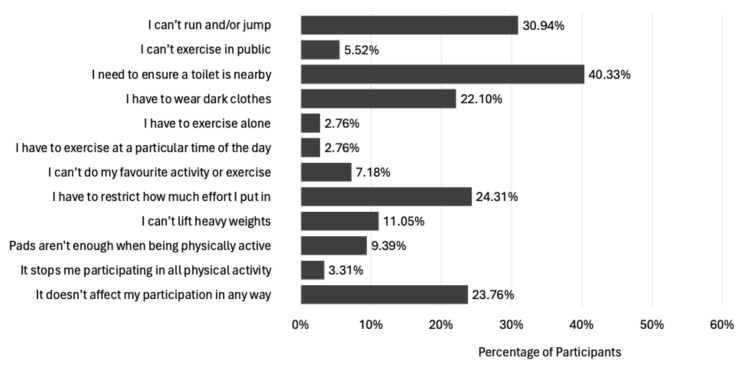
Effect of UI on women’s PA participation (n = 188); multiple responses could be chosen.

With nearly two-thirds of women experiencing UI during PA, it seems prudent to identify the main ways leaking urine affects PA participation. The findings illustrated in Figure 5 align with those of previous studies, though these tend to be framed within a ‘coping strategies’ context. By framing the question posed as ‘effect on PA’, it seems the change in context captured some lesser reported effects. For example, Brennand et al. [19] found nearly half of women wore some form of pad to help contain leaking during PA, and, although they explored the type of pad and whether it caused irritation, they did not appear to capture data on women’s strategies when pads failed to provide adequate protection. For women in the current survey, 9% reported pads alone were not enough to contain leaks during exercise, likely increasing the risk of withdrawal from PA if no other strategy could be found to work.

Brennand et al. [19] also reported 93.2% of incontinent women void their bladders before exercise commencement, and only 37.3% manage to undertake a full exercise session without taking at least one toilet break. These findings are commonly reported in the literature; for example, see Chisholm et al. [55]. Similarly, participants in the current study also reported toilet location as a concern, with almost 40% of participants stating a need for access to a toilet close to where exercise was being performed. This need is supported by a recent study that found some women had swapped large gyms for smaller ones to ensure toilets were closer [21], suggesting women’s needs concerning UI and toilet provision may need additional consideration at the planning stages of designing PA venues. The need for accessible toilets close to where women participate in PA may be a particularly important point, given the current propensity to design ‘Low Emission Zones’ and ‘Active Freeways’ in many cities, with the aim to encourage people to make use of green spaces and promote active travel. It seems that any failing to consider UI’s impact on PA participation and not consider providing adequate and convenient toileting opportunities may limit the opportunity to engage women in such schemes successfully. Considering toilet provision and women’s needs for managing UI during PA when designing active travel infrastructure at the planning stage is even more important, given previous research has shown the availability of safe cycling lanes and public transport are more strongly associated with women’s PA engagement than men’s [56]. Women also travel shorter distances more frequently, and these are less for leisure and more often for domestic and caregiving reasons [57]. Thus, failing to consider the toileting needs of women in active travel schemes would seem to overlook the very population most likely to benefit and engage with such schemes.

#### 3.1.5. Being Asked to Disclose Symptoms by Others

**Figure 6:**
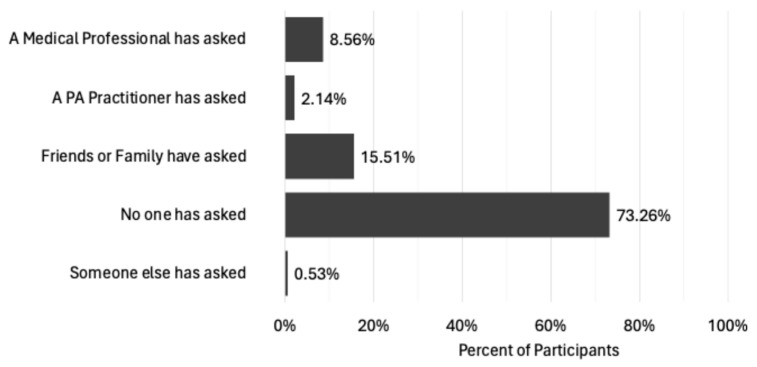
Frequency of being asked UI status (n = 187).

As previously stipulated, PAH practitioners should not rely on women disclosing UI as a barrier to their PA spontaneously. To enable practitioners to confidently enquire about a woman’s UI experiences, it seems prudent to gain an understanding of women’s comfort levels regarding their UI status being questioned. Participants were first requested to indicate if they had been asked at any time in adulthood whether they experienced UI during PA (Figure 6). Disappointingly, over 73% of respondents had never been asked if they leak urine during exercise by anyone, and whilst 8.6% had been asked by a medical professional, this was eclipsed by friends and family (15.5%). Whilst recent research suggests PA practitioners are mostly comfortable discussing the pelvic floor with exercisers [58], it also suggests they are not asking about pelvic floor status, preferring to wait for the individual to raise it as a concern to them first [59]. It was therefore heartening to see that at least a few women (2.1%) had been asked about their UI status by a PA practitioner.

**Figure 7:**
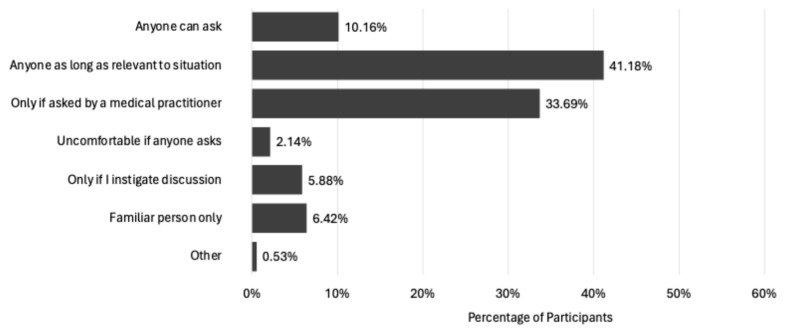
Comfort with being asked UI Status (n = 187).

Next, participants were asked to indicate their comfort levels at being asked by others to disclose their UI status. The findings indicate that 41.2% are happy to be asked their UI status by anyone if it is relevant to the situation, whilst 10.2% were happy for anyone to ask regardless (Figure 7). However, 33.7% indicated they are only comfortable if a medical practitioner asked, and 6.4% were not comfortable with disclosing their UI status, regardless of who the person was that asked them. In addition, almost 6% were not comfortable with any form of enquiry and would prefer to raise the condition in discussion themselves if they so wished.

Women’s comfort levels with disclosing their UI status to others appear varied, which may be concerning for practitioners screening for the condition in the PA environment; however, at least 50% appear to be open to enquiries when UI treatment or advice is provided. Additional factors which could be implemented to help increase women’s comfort at being asked their UI status. For example, in the clinical setting women are reported to be more comfortable discussing UI with a female clinician [60], and some studies have found a preference to be treated by female clinicians is especially significant in individuals with UI compared to other urological conditions [61,62]. It therefore could be assumed that the women in the current study would be more forthcoming with their UI status to a female practitioner. However, 50% of participants reported having no preference for the sex of the person asking about their UI status, while 50% stated they would prefer a female practitioner. ‘Male’ was an option; however, no participant reported a preference for a male practitioner when discussing their UI symptoms.

These findings are similar to medical studies that have reported between 54% and 80% of patients had no preference in the sex of their urology clinician [62,63]. Requesting a clinician of the same sex is largely attributed to the level of symptom embarrassment felt and a belief that personal experiences of the condition will be better understood by someone of the same sex [61]. However, it is unclear from the study findings whether women in the PA environment choose a female practitioner to disclose their condition to for the same reasons, and this warrants further investigation to clarify.

These survey findings suggest UI has a notable impact on women’s PA participation, but it may be underreported as a barrier due to women’s reluctance to self-disclose symptoms unless explicitly asked to consider the condition. It is clear many women are comfortable being asked to disclose their UI status in the PA environment; however, gaining a deeper understanding of the circumstances in which women are comfortable to disclose, and developing a better understanding of the preferred methods for obtaining UI status information, such as through questionnaires or verbally, may help reluctant PAH practitioners to have the confidence to tackle UI as a barrier within their PA practice. It also seems pertinent to elaborate on the findings of this survey by exploring the responses in more detail. For these reasons, the findings of the survey were used to inform the questions of the interview phase of the study (phase 2).

### 3.2. Phase 2 Interview Findings

Fourteen women were interviewed with the aim of understanding women’s feelings and perceptions of UI disclosure inside the PA environment and to understand women’s experiences of UI during PA. Thematic analysis uncovered 3 general dimensions: Forced Behaviour Change, To Disclose or Not Disclose, and Encouraging Continued Participation.

#### 3.2.1. Forced Behaviour Change

- Negative Emotions

During the interviews, the participants often referred to the impact of UI on PA in terms of the negative effect it had on their emotions, with all expressing feelings of frustration or anger. Several of the women felt these feelings mainly came from their sense of wanting to get fitter, but experiencing leakage was holding them back:

*“I think when I’m exercising it is that frustration of doing this because I want to get fitter. It’s frustrating, and it does make me feel angry, but I think what frustrates me the most is that the whole time I’m doing any exercise I’m always thinking about wetting”.*

Participants felt the constant awareness that they were likely to leak urine during workouts was the most frustrating aspect. Several participants displayed a high motivation to exercise and being healthy appeared a high priority for all the women interviewed. However, achieving or maintaining motivation was expressed as being significantly more difficult due to the presence of UI, as the above quoted participant further clarifies:

*“I think it’s that I know I’m capable of doing more, but I don’t, because I don’t want to leak, I don’t want to be embarrassed and don’t want to feel horrible. So, I kind of feel like I’m doing half of the work that I should be doing and that I could do more”.*

Women with UI are known to reduce their effort during exercises that carry a high risk of leaking as a strategy to continue exercising and self-manage urine loss during participation [21]; however, this can result in increased frustration, negatively impacting motivation, as illustrated by the participant. A lack of motivation is an often-reported barrier to PA [13,50], and although regularly targeted in PA interventions, often through the implementation of strategies such as goal setting [64], UI appears to present an additional challenge for intervention design. Furthermore, motivation may also be negatively affected by feelings of increased exercise anxiety which experiencing UI during PA can invoke. How much interviewees were ‘bothered’ by their experiences of leaking urine during PA seemed varied; however, all alluded to UI making them anxious during exercise participation to some degree. Predominantly, the unpredictability of leaking seemed to be the main contributor to exercise anxiety, and even where leaking was slight or seldomly experienced, an increase in anxiety developed from simply knowing leaking was a possibility. The main outcome from these experiences was the willingness to continue exercising diminished, and for some, it resulted in withdrawal from some or all types of PA participation:

*“I’m worried about it happening when it happens, after it happens, and it gets to the stage where you just think, ‘Oh, I just don’t want to do it’”.*

For those participants who continued to engage in their favourite mode of exercise, anxiety during PA appeared less of a concern than for those who had to change modes due to leaking. However, for many, the anxiety over whether they would leak appeared to begin as soon as they considered attending a PA session. For one participant whose preference was participating in social forms of exercise, anxiety would begin to develop from the moment a friend requested she join her for an exercise session:

*“It’s the number one thing. I feel it even before I start the exercise, the anxiousness of ‘Oh God, is it actually going to happen’? You know, ‘will I get through this type of exercise without leaking or not?’”.*

Several of the participants confirmed that evaluating the risk of leaking was a particularly prominent consideration during their PA planning process, and often feelings of anxiety would begin immediately upon this process starting and continue whenever they thought of their arranged activity, only lessening once the PA session ended. In the absence of strategies to bolster their confidence that leaking could be managed in front of others, many women reported their reluctance to consider any further social PA attendance.

Whilst research has provided evidence that sedentary behaviours increase the risk of anxiety in women [65], including during menopause [66], a correlation has also been reported between high severity of UI and anxiety prevalence [67], compounding any treatment of anxiety through PA prescription. Whilst the causal relationship between UI and anxiety appears uncertain, these findings suggest a need for PAH practitioners to be aware of this relationship to effectively mitigate the risk of anxiety development during PA participation.

Perhaps somewhat expectedly, a loss of enjoyment was the resulting outcome for many participants feeling anxious in the PA environment or feeling frustrated at the lack of perceived effort they were able to exert; however, these were not the only circumstances women reported as leading to PA enjoyment loss. Several women reported a failure to engage with others during social exercise sessions due to the necessity of maintaining concentration on their bodies to avoid leaking, which meant they could not interact with others effectively:

*“I’m so focused on what’s happening in my body that I feel if I lose that concentration by talking and engaging and being present in the class, my concentration breaks on holding it all together and holding it all in. So, you know, you lose the enjoyment”.*

For some, their focus was on contracting their pelvic floor muscles, which allowed them to continue performing specific exercises and limit leaking, whilst others suggested their concentration was directed at detecting the slightest feeling that a leak was imminent, allowing for swift remedial action to be taken, such as exiting the session to visit the toilet. One participant also reported her concentration was mainly used to assess each exercise instructed by the fitness leader to help her evaluate the chance of undertaking the movement without incident. In all cases, participants felt that UI reduced their opportunity to socialise with others and enjoy their PA session.

Enjoyment has been shown to be a particularly important factor to target in PA interventions, as it has been shown to improve PA motivation and exercise maintenance [68]. Older women have reported experiencing greater enjoyment by participating in group physical activities, especially as they value opportunities beyond the home for socialising, often stating being held accountable by others is their motivation for attendance [69,70]. These findings identify enjoyment as a possible mechanism for manipulation in interventions aimed at increasing women’s PA levels. When considered alongside the literature findings, these combined findings may suggest that any PAH practitioner who fails to consider UI’s impact on PA participation in their intervention design may find themselves thwarted from successfully employing ‘enjoyment’ as a motivator to improve women’s PA participation.

- No Choice

Participants overwhelmingly reported that they felt UI forced them to modify their PA in some way, with many particularly bemoaning that UI contributed to the relinquishing of a favourite exercise mode. Some participants who felt they had been forced to change PA modes were able to maintain their PA levels; however, the majority reported it contributed to a loss of enjoyment which resulted in a drop in PA levels or, for at least two participants, complete withdrawal from all PA. As one participant explained, she would prefer to participate in a different mode of exercise but, through trial and error, has found a mode which results in minimal leakage, helping her maintain her PA levels:

*“I don’t do the exercise I want to do, I do the exercise I’ve worked out I can do. And that’s the thing for me, that I would do more, and I would do different, but I don’t trust myself, and I don’t want to be in an awkward position”.*

This participant’s favourite mode of exercise was a local group fitness class, and most participants seemed to lament the loss of group PA particularly. The popularity of group PA expressed by the participants is confirmed in the literature [71]; however, there are surprisingly few studies which have looked at the contribution UI makes to women withdrawing from this mode of PA specifically. Of note, a systematic review by Dakic et al. [59] confirmed the withdrawal of group exercise by women due to UI, further stating withdrawal is particularly likely where the group is mixed sex. Whilst interviewees in this study did not spontaneously present a group member’s sex as a contributing factor to their withdrawal, being fearful that others in a group setting would smell urine if they leaked appeared to be a common concern:

*“That’s another barrier for me, the fact that can people smell me? The fact that I’m incontinent. It frightens me that other people think I smell”.*

Although almost all participants interviewed spontaneously raised the concern of others perceiving the odour of urine from them, it has been barely alluded to in PA studies [20,72]. Fear of the odour of UI appears to be discussed more in studies looking at UI in general. For example, a qualitative study exploring the experiences of men and women living with UI found participants worried they smelt, and the authors linked this with concern over a loss of bodily control [73]. Another study looked at Norwegian municipal workers and the impact of UI on their work capacity, finding that participants worried about UI odours daily and that this affected their work-related QoL [74]. Southall et al. [24] assert in the introductory section of their study evaluating UI intervention measures and stigma content that withdrawal from social engagement due to UI is stressful for individuals, asserting that this may be due to the presence of urine smells hampering the individual’s ability to hide UI from others. Indeed, some interviewees suggested they would not so much as consider taking part in group PA interventions in the future due to this fear which, it may be concluded, indicates that a concern over the smell when leaking urine contributes significantly to the barrier UI presents to women’s PA participation.

Withdrawal from group exercise was further discussed by participants with little probing by the researcher, with all intimating to some degree that the main reason they preferred group exercise was due to the opportunity it presented for socialising. Although the socialising aspect was not explored further, it became clear that most women put a great deal of effort into trying to avoid relinquishing this particular favourite exercise, firstly by attempting to find less-problematic types of group PA:

*“I started trying different things like, could I do a keep-fit class? Or could I do a HIIT class or something? And I couldn’t. I wasn’t getting through any of them”.*

Trying different types of fitness classes in the hope one would keep leaking at a manageable level seemed a common approach for those determined to remain in group exercise. Whether participants sought out alternative modes of group PA did not seem to be dependent on whether they had high levels of participation historically. Of the fourteen women interviewed, only one indicated she had successfully found a suitable group to attend. The majority, however, once they had experienced their first bout of leaking during a class, had been too fearful of further leaking episodes to even consider attempting exercise in front of others:

*“There’s a few classes that have popped up locally and I’ve thought ‘Oh, I’d quite like to do that’, and then I’ve thought ‘mmmm, probably just be getting myself all wet and...’, you know, that just puts me off, and I just think nah, I don’t want to do it”.*

Statistics regarding the level of withdrawal from group exercise could not currently be found in the literature, possibly due to most UI/PA studies excluding women who have already withdrawn from PA, and as such, we have little idea of the rates of withdrawal. However, we do know that exercise levels reduce in midlife women and that a feeling of being accountable to others is one of the most reported factors to aid the maintenance of PA participation in this population [75], although more recent research suggests the social benefits of exercising in a group may improve motivation more than feeling obligated to others [76]. Regardless, if group PA appears to be a mode women enjoy and are motivated to participate in, it may be prudent to find a way for women to remain participatory in group exercise after the onset of UI.

Where women could not continue participating in group PA due to the risk of leaking, they often searched for alternative low-impact ways to continue being active; however, these alternative modes could also be a challenge due to the presence of UI. Of those women who reported substituting their favourite PA mode for an alternative to try and remain active, walking seemed to be thought of as universally ‘safe’ regarding the risk of leaking urine; a view confirmed in a study by Bauer et al. [77] which concluded that high levels of PA, and particularly walking, were associated with a lower risk of UI, though significance may change depending on UI type and severity. Walking also helped those who reported additional barriers to exercise to contribute to their PA levels. This was particularly true of those who reported barriers to their exercise from caring responsibilities or work commitments, as these participants appeared to suggest that walking for PA was more of an ‘opportunistic’ endeavour that could be undertaken as part of their domestic or work chores. Almost exclusively, they would opt to walk to/from work, to/from the bus stop, or to/from the shops to reach their PA goals. However, whilst switching to low-impact exercises like walking could be a successful strategy for many, some participants reported UI could still create disruption to ‘suitable’ alternative PA modes:

*“Where I live, it’s about a mile and a half into town. I walk into town providing that my first stop has got a loo. Even in supermarkets before I set off home, I’ll think about if I need to use the loo”.*

Many women felt a great deal of consideration and planning was required to include walking in their domestic and social life, but with many towns and urban areas losing public toilet provision [78], some women felt it was very difficult to achieve success. In addition, the unpredictability of incontinence also seemed to create an additional burden which could often result in the curtailment of a walk:

*“Sometimes I think I’m perfectly fine, I’ve not had an incident, and then I’ll just walk up the street five minutes, and then it’s running down my leg and I’ve got turn around and come back”.*

It appears that despite changing their exercise mode to a lower intensity one, a risk of leaking can still occur for some women. For women who view walking as their last option to remain physically active, continuing to leak during exercise appears to be particularly distressing and may lead to complete withdrawal from all PA. In addition, some participants voiced that they would be unwilling to consider any future PA participation, regardless of the mode or intensity. Indeed, this scenario was described by two women who were currently inactive; however, another participant who stated she had maintained high levels of PA across her lifetime until UI occurred felt determined to not allow leaking during walks to stop her:

*“It’s a mental barrier I have to overcome to even do anything. I think it’s because I was so active and fit before, where I was the one at the front leading everything and being the one that everybody looked to and now, I hide away and I’m not able to manage [exercise]”.*

The participant, who was in her early forties, was extremely angry that UI stopped her achieving her PA goals, particularly at such a young age. She referenced being naturally gregarious and fun-loving prior to experiencing UI, but when the discussion moved to her post-UI experience, a clear change in verbal and body language was observed. By the end of the interview, it was clear UI had had a profound effect, and the participant was far more introverted as a result. Her self-esteem appeared low, and she seemed very frustrated and saddened by her experience. Many other participants were also observed to have experienced a negative impact on their self-esteem due to UI, albeit to a somewhat lesser degree.

Low self-esteem seems common in women with UI and, as a result, has been well researched in QoL studies, though less so in the context of PA. In their review, Gümüşsoy, Kavlak and Dönmez [79] found that women with UI had low to moderate self-esteem, which was worse in those over 50 years of age, and a non-UI study reported mid-life women with high levels of PA also had high levels of self-esteem [80], suggesting that whilst UI contributes to women’s low self-esteem, PA may be a suitable pursuit to counteract its effects—of course, that is as long as suitable strategies can be found to help women manage the risk of UI when doing so.

#### 3.2.2. To Disclose or Not Disclose

- Reasons to Disclose UI Status

The main aim of the phase 1 survey was to test whether women would make an unprompted disclosure of UI symptoms when questioned about the barriers they experienced to PA. As such a small number of participants (n = 4) spontaneously listed UI in their response, it was important to include as many of these respondents in phase 2 as possible. All four women who listed UI as a barrier to their PA unprompted agreed to be interviewed and are included in the interview findings.

For all but one of the four women, having to change their exercise mode to something less coveted appeared to be the main reason for unprompted disclosure, although it did not seem as straightforward as simply being annoyed:

*“Why did I write it? Because wetting myself stops me doing stuff. I mean, I belong to a gym, I’m okay going on the machines because you wipe the machines down afterwards and everything, but I can’t do spin or anything like that”.*

The participant expressed her annoyance at UI impacting her ability to participate in the type of exercise she desires. Her frustration was palpable, and she voiced her preference for spin classes more than once, feeling these were impossible to attend because the intensity would increase her leaking and she would have to wipe down the bike seat in front of others, which would be embarrassing for her. Thus, she was restricted to working out on gym machines where she could disguise any need to wipe down the seats as the more socially acceptable and less stigmatised practice of wiping away sweat. Most of the women who disclosed UI unprompted described ways of coping with UI in the PA environment in a similar way; however, trying to hide a condition, particularly one which is viewed negatively by society, such as UI, by disguising it as a more accepted condition is called “passing” in stigma theory and can be a way for individuals to continue to function in society, hiding their condition from others and appearing “normal” [81] Continually having to find ways to hide UI can be exhausting, and, as Goffman [81] suggests, this can lead the individual to seek out places where they can be themselves and, usually where others like them can be found. In the modern day, this may be a UI support group, for example, and it may be that the current study provided these women with a similar opportunity to cease hiding their UI condition, feeling that they could be themselves and not be judged for revealing UI as a barrier to their PA. Previous research has confirmed that whilst a fear of being stigmatised can stop women disclosing their condition [73], disclosing UI symptoms to others may also be a way to combat stigma and reduce that fear [47], and providing the opportunity to disclose may be a useful strategy to incorporate in intervention design to further encourage women’s engagement.

However, more obvious in the above quote, and in all four participants’ discussions, was their frustration at having to change their PA practice because of leaking:

*“Because it’s a serious issue for me in the sense of you know, I think when you’ve been very fit and then all of a sudden to feel the mental health impact of not exercising, you know, and then trying to adapt your practice to cope with that”.*

For all four participants who voluntarily disclosed UI as a barrier, there was clear indication that the impact of UI on their QoL was a driving factor. For many of the women interviewed, being forced to change exercise mode or volume impacted the health, leisure and social goals they set themselves. The negative impact of UI on QoL is well reported in the literature, and both those with low QoL and those with more severe UI have been shown to have higher levels of anxiety and depression [67], as has been mentioned earlier in this section. However, individuals who do not achieve sufficient PA have also been shown to have lower QoL and higher anxiety and depression [65], thus making the negative impact of UI on PA all the more intolerable for those women who have had to reduce their participation. It may be that these compounding factors result in individuals becoming so overwhelmed in their attempts to remain active that they become desperate for help and disclose their UI status at any opportunity, in the hope that help is forthcoming. This assertion is evidenced somewhat in the literature by Fakari et al. [23], who found that people with UI who perceive their symptoms as impacting their QoL were more likely to disclose their condition and seek treatment. A more recent study also supports these findings by reporting much lower QoL scores were found in women who had sought help for their UI, compared to those who were yet to do so [82].

- Reasons for Non-Disclosure of UI Status

It is clear from the testimony thus far that the severity of UI’s impact on QoL is dependent on the severity of UI’s impact on the participant’s PA, and the combined force of both seems to be the main catalyst for the four participants disclosing their UI as a barrier, unprompted. As the survey shows, when prompted, a total of 254 participants reported UI as a barrier to PA, which may lead to the assumption that the other participants did not perceive UI’s impact on PA as severely as the women responding without prompting, yet participants seemed to suggest otherwise:

*“I think it’s because you start to think, well, I really don’t want it to be a barrier. It is, but I don’t want it to be. Embarrassment would be why maybe I didn’t write it down as a cause. But I am aware that it is my number one cause, the leaking, and the urgency for needing to go to the toilet. I am aware that that is the problem for me with a lot of things. I mean, I’ve got a skipping rope. I’ve got a hula hoop, and they’re things that, just at this moment in time, I cannot cope with”.*

The participant’s symptoms are so severe that she is unable to participate in the exercises she has equipment for at home, and she describes it as her “number one” barrier. Yet, the UI was not disclosed as a barrier until the participant was specifically asked to consider it. In her reasoning, she suggests that she may have been too embarrassed to consider disclosing the condition and seems dismayed that she feels this way. Embarrassment is mentioned regularly by most of the participants interviewed and is well reported in the UI literature. For example, a systematic review found that even women undertaking low-impact exercise, such as swimming, had withdrawn from PA due to embarrassment and the fear they would leak [83]. In another systematic review on help-seeking in women with stigmatised pelvic-floor conditions, embarrassment was found to be one of the most reported barriers to women disclosing their symptoms to a health professional [84], and another, looking at pelvic floor dysfunction in athletes, found 6 out of 9 studies in their review reported embarrassment was the main emotion negatively affected [20]. If UI can be discussed in the PA environment, it would seem it has the potential to fight stigma and help alleviate the emotional burden women are experiencing when trying to be physically active.

Another participant also reasoned that embarrassment stopped her from disclosing UI as a barrier unprompted:

*“I think it’s still embarrassing. I think sometimes you try and you kind of try and put it to the back of your head and I think then if someone specifically asks you, you kind of go ‘Oh well, yeah, I suppose so’”.*

Whilst the participant tried to just ignore it and so did not think to respond until prompted. Whilst explaining her reasoning, it seemed she was unmotivated to participate in a type of PA that was not her first choice. She mentioned several times about her historical love of running and that now, all she could manage was Pilates and similar low intensity PA modes, and these did not seem to be meeting her psychological health needs. Within self-determination theory is basic needs theory, which suggests that for people to thrive, basic needs satisfaction is fundamental, specifically in regard to the needs of competence (i.e., to feel able and capable of meeting challenges), autonomy (i.e., freedom to make decisions and choose), and relatedness (i.e., the need to connect with other people) [85]. When the individual feels these needs are being met, good well-being is exhibited and higher internal motivation to continue often results [86]. However, for the participant quoted above and several of the others, it was clear that the reduction or cessation of PA or being forced to change the type of PA undertaken impacted some or all of these basic psychological needs, resulting in their mental health and internal motivation being negatively impacted. Previous studies have shown no negative effects on PA when all three of these basic psychological needs are met [87]; therefore, if women could be supported in managing their UI during PA or be helped to strategise alternative modes of exercise that continue to fulfil these basic needs, perhaps reductions in motivation and mental health could be avoided.

For other participants, their non-disclosure of UI as a barrier unprompted was because they perceived it as unimportant compared to other health conditions:

*“It would have become one of my top 10 but it is the fact that although it’s very important it’s top-trumped by other physical issues”.*

It is well known that women live longer but are more likely to experience poor health, disability and frailty as they age compared to men [88]. As the risk of developing many conditions, such as stroke and osteoporosis, increases with age [89], it seems likely that women from midlife and upwards will experience one or more comorbidities alongside UI [90]. It is known women delay seeking medical treatment for UI because other conditions take priority, and it appears PAH practitioners need to be mindful that this may also be a reason women with UI do not disclose their UI unprompted. Indeed, upon analysing the barriers the quoted participant listed unprompted, there were several physiological conditions listed which may impact this participant’s ability to engage in PA more.

- Comfort with UI Status Enquiry

After exploring the reasons why participants did or did not disclose UI as a barrier to PA unprompted, it seemed pertinent to understand how comfortable the participants were with being asked to divulge such sensitive information, as well as what factors could be implemented by PAH practitioners to further improve their comfort in responding to such an enquiry. Given that 50% of the survey participants indicated they would prefer a female instructor to ask them about their UI status, this discussion began by exploring the perceptions the participants held on the sex of fitness instructors.

A recent study by Hebert and McGuin [91] reported that their mixed-sex participants (n = 144 female, 57 male) perceived male fitness instructors more positively than female instructors, especially when working with them for long periods and in regard to having more fitness knowledge than their female counterparts. However, they also reported that female instructors were perceived more positively for discussing any problems they were having with their PA participation [91]. Findings, however, are mixed; an earlier study found no sex preference for fitness instructors by their male or female participants [92], but for the current study’s participants, it seems that sex and age matter when instructors are questioning women about their UI status:

*“The fact that they were asking a question about it, presumably so that they could tailor their class to suit me, would be a good thing but I have to say, I would be slightly less comfortable if it was a man”.*

Preferring a female fitness instructor when discussing UI is echoed in the literature regarding disclosure in the clinical environment, where participants with unspecified urological conditions expressed no preference towards their medical practitioner’s sex, but when only those experiencing UI were asked, they were found to have a significant need for same-sex practitioners [61].

For two participants, the age of the participant seemed equally important, but for another in particular, a male instructor who was also of a younger age (teen or early twenties) was enough of an issue that the participant would be unwilling to consider responding to any enquiry about UI:

*“If it was somebody I didn’t know, and particularly somebody younger, and particularly somebody male would I? Well, I’m not sure what I would actually. I think I’d probably lose my nerve and lie”.*

The quoted participant had withdrawn from group exercise due to pads failing to control her leaking, choosing instead to exercise at home. She mentioned the age of PAH practitioners, irrespective of their sex, several times during the interview and seemed quite concerned about being faced with a young instructor. Whilst not explicitly stated, the researcher felt that any risk of UI enquiry, regardless of the motivation behind it, would end in the participant withdrawing from that space entirely; however, no real explanation was forthcoming regarding why the instructor’s age would cause such an issue. The literature also did not seem to contain any answers on this matter.

Whilst the previously mentioned study by Hebert and McGuin [91] found that perceptions of male fitness instructor knowledge were more favourable than female instructors, the opposite appears true when instructors are discussing knowledge of UI:

*“I’d be less likely to go to a man. I don’t know. I just feel like they don’t understand it. And maybe this is where I see it as a female problem. Maybe it’s not just a female problem but I think because of that, it’s in my head, they really don’t get that you’ve no control over this. So yeah, no, I would much rather a woman asked”.*

The assumption that a female instructor would be more likely to understand the impact of UI on PA behaviour and women’s comfort levels is understandable; one would surmise this may be particularly true if the instructor personally experienced UI too. This is supported in the limited research conducted on PA instructors experiencing UI, where the prevalence rate of UI in female Pilates, yoga and group fitness instructors was reported at similar levels to the general female population [93]. The same study also reported the incidence of UI increased in the fitness instructors with age and this may help to explain the so far unanswered question of why some of the current study participants voiced a preference for older female instructors—the participants believe this demographic are more likely to also experience UI and therefore understand UI as a barrier to their PA.

Even with an older, female instructor enquiring about their UI status, some participants were still not entirely comfortable and seemed skeptical of practitioner motives for asking:

*“If she [the instructor] seemed quite knowledgeable about it and framed it in such a way that we understand that some people can have problems with this and we can adapt our exercises to suit that. Or to even maybe target the muscles that you need, that would probably make me more comfortable that it’s not just a sort of tick-box, and they’re actually going to be able to help me”.*

Several participants appeared sceptical of any fitness instructor’s ability to provide practical help that would improve their PA outcomes. This assertion may be well-founded, given no published studies could be found to suggest fitness instructors have tested knowledge of how to instruct pelvic floor muscle contractions correctly. There are, however, several studies which recommend pelvic floor muscle training (PFMT) be made available in the fitness or non-medical setting. For example, Bø [94] advises that both coaches and fitness instructors have a role to play in helping exercising women and athletes in improving their continence, whilst another study reported that fitness instructors in the gym environment would be happy to deliver PFMT instruction so long as they were adequately trained to do so first [58]. Finally, Scime et al. [90] Brubaker et al. [95] successfully illustrated the feasibility of providing PFMT instruction and education classes delivered by non-medical instructors in the PA environment, which suggests that whilst little instruction is taking place currently, providing PFMT instruction in the PA environment may help facilitate first-line healthcare for women with UI. It should be noted that some fitness instructors report including pelvic muscle engagement cues in their fitness instruction; however, Bø [94] suggests that it is unknown if these are correctly instructed pelvic floor muscle contractions or little more than an acknowledgement to ‘lift and squeeze’. However a more recent study found that almost half their female participants could not perform pelvic floor muscle contractions without detailed instruction, indicating a necessity for fitness instructors to receive adequate training on pelvic floor exercises from a pelvic health professional before instructing women.

The final finding in the ‘Comfort with UI-Status Enquiry’ theme was that the response format has influence on comfort and was spontaneously raised by several participants at different points in the interview. The first participant to mention that they would be more comfortable if they were asked about experiencing UI via a ‘joining’ questionnaire in a gym (for example) did so whilst discussing her preference for a female instructor:

*“I think it would be important for it to be on the questionnaire. So that then there could be a verbal conversation with the PT based on your answer”.*

The participant felt that responding to an open-ended questionnaire would allow for additional detail to be added if they felt it was relevant and ensure a conversation could be had about how best the instructor could support the participant. Being asked in a questionnaire also seemed favoured by the participant who expressed the most discomfort at any enquiry of her UI status, although she was very clear to explain her preference for checkboxes:

*“If there was multiple choice components, like, if you have, have you ever experienced the following? And it’s there, then people are more likely to check that answer. Especially if there’s multiples because then they feel like, ‘Oh, I’ve said it, but it’s not blaringly flashing’, that that’s the thing”.*

For the more uncomfortable participant, a checkbox seemed to offer a less confronting way to acknowledge a sensitive condition such as UI, and she appeared willing to confirm her condition that way. However, when asked if she would be happy for the practitioner to then start a conversation about how UI impacted her exercising, the participant looked alarmed and shook her head before re-stating that it would ‘depend on who the practitioner was’.

The usefulness of questionnaires in the gathering of sensitive data has been described in the literature, although most contemporary papers purport the efficacy of web-based questionnaires. Braun et al. [96] recommend qualitative surveys for very sensitive data, but when paper and online questionnaires were compared, researchers found a higher rate of non-responses on the paper questionnaire overall, and particularly so with the most sensitive questions [97]. It may be that for the most sensitive women there is an increased chance that questions asking about their UI status may go unanswered, and further research is needed of how best to screen for UI in the PA environment.

#### 3.2.3. Encouraging Continued Participation

- Updating Exercise Delivery to Meet All Women’s Needs

The final dimension of the study seeks to understand ways in which PAH practitioners can encourage women with UI to maintain or improve their PA participation using participants’ own suggestions, expressed at different times throughout the interviews. The first suggestion identified was the need for instructors to offer better in-class move adaptations, as one participant explained:

*“You’ve got to be able to adapt that lesson to lots of different people, and I just don’t think that there’s the understanding and the training in fitness to actually understand that at the moment. I just, I really don’t”.*

The lack of PA practitioner knowledge on effective PFMT instruction has been previously discussed; however, the lack of practitioner insight into, or consideration of, the ways in which UI impacts women’s ability to successfully accomplish specific exercise moves was a particular source of frustration for the most physically active participants. Without understanding, these participants felt fitness instructors were unable to provide alternative moves that would allow them to fully engage in a group class, as another of the most active participants clarified:

*“They’re not going to be judgmental if you’ve got to nip off for a wee. And they’ll work around that. They’ll probably not put as many star jumps in and not do things that are going to cause people to leak. They’re aware of what can make a lady wee herself, or whatever”.*

Having that insight, they hoped, would mean that instructors would remove problematic exercise moves, such as “star jumps” (which, incidentally, featured heavily in many interviews). Unfortunately, there is little research examining adaptations to exercise moves by fitness instructors; most papers looking to explain the impact of UI on women’s PA behaviour do cite exercise adaptations as one of the strategies women themselves employ to avoid leaking during PA. For example, Brennand et al. [19] reported 90% of their participants decreased the intensity of exercises performed, and almost 81% avoided specific activities to minimise leaking. However to fully encourage women with UI to continue in group exercise, exploring ways for instructors to provide adaptations in class would help those women who particularly covet group PA to remain engaged and motivated.

Another participant was very keen to point out that she withdrew from group exercise not due to leaking so much as due to the frustration of being perceived as lazy:

*“I want to do that, but I can’t and I’m not going to stand in the middle of an exercise class and tell you I can’t do a star jump because I’ll wee myself. Can you give me something that’s as energetic but takes into account the fact that I might not want to do that, not because it’s harder, I just don’t want to do a star jump”.*

With a high level of motivation for PA, this participant alluded to PA achievements throughout her interview, indicating that pushing herself to work hard and achieve her PA goals was important to her. As such, being perceived as lazy or incapable was felt as ‘failure’. She felt that her group instructor viewed her as lazy because she would not attempt star jumps when they were programmed into the class routine and that it was assumed star jumps were ‘too hard’ and she was not good enough to do them, rather than the truth that she would leak in front of the class if she attempted them. As mentioned previously, competence, or the need to feel capable of meeting challenges, is one of the three key needs in basic psychological needs theory (part of SDT), and the participant’s inability to overcome others’ perceptions of her was having a negative effect on her psychological needs, which, in turn, impacted her motivation to continue with group PA. Finding a way for instructors to adapt specific problematic moves in such a way that doing so minimises women’s feelings of failure or others’ perceptions of their effort may help meet similar women’s needs better.

One of the most popular adaptations suggested by participants was to introduce classes that had an obvious aim of helping women with UI improve their symptoms by incorporating PFMT instruction into classes. However, the participants who had expressed little desire to disclose their UI status earlier in the interview process pleaded that there be ‘no dedicated classes—please!’:

*“If they’re just doing general PT so that there could be someone in there before me or after me, and if they see me, they just think, ‘Oh, she’s in there for PT’, and there’s no [risk] of ‘She’s in there for incontinence!’”.*

For those women, the thought of a dedicated UI specific fitness session was outside of their comfort levels because they feared being observed entering or exiting such a class could result in their condition becoming known by others. These feelings are generally born from the fear of stigma, where others finding out could result in humiliation and judgement [47]; however, other participants felt that a clearly advertised class would positively affect their comfort with others discovering their UI status:

*“I think if there was a clear thing that said that leaking was taken into consideration, then I think I think I probably would feel more comfortable because I’d feel that the person who was taking the group actually had an understanding about it and would actually then probably discuss it with you”.*

Attending a UI dedicated class afforded a certain level of trust to the group’s instructor that had thus far been absent from the conversation. Advertising a class as being suitable to those with UI may provide confidence that they would not be judged or humiliated and their needs will be met to enable them to fully take part in the class. Additionally, some participants felt that a dedicated class, or one that at least advertised itself as being mindful of the needs of urinary incontinent women, could also help with the third basic psychological need—relatedness, or the need to connect with others:

*“I think it would make a massive difference because you go to classes, and I think you feel quite isolated because you look around and you think everyone else has got their shit together and I’m kind of standing there concerned about the fact I’m not doing half the exercises. I don’t feel that I can. It just makes you feel like not going back”.*

Feeling alone in experiencing UI is common, as women withdraw from social situations to try and avoid others discovering their condition [60,73]. However, for almost all the participants except the very fearful, attending a group class where they knew others with the same condition and similar experiences would be gave them confidence to participate and be themselves.

Unexpectedly, two participants independently raised the point that there may be a role for humour in UI education:

*“I think the thing is, without female comedians, you wouldn’t find any of this out. If you go to see some of the people that, I don’t know if you’ve ever seen any female comedians do the menopause? But you walk out of there thinking ‘crikey! I didn’t know that!’. You’re in a roomful of hormonal women who’ve lost most of the hormones and they’re all sighing a sigh of relief”.*

In the above example, female comedians are perceived to take on the role of educator and are exhibiting many of the demographics the participants had expressed as desirable in PA instructors, such as possessing knowledge that can help participants manage their condition and having first-hand personal experience of the shared condition, leading women to feel the comedians can empathise with them. There is a small but growing body of evidence suggesting that humour can be an effective tool in engaging individuals from hard-to-reach population; however, there are some limits to humour’s success. For example, a study which framed posters advertising health messaging for different health conditions with humour reported posters with health messaging for advanced care were positively received by participants, whilst others, including cancer screening, smoking cessation, and PA messaging, received lower scores [98], suggesting care is needed if humour is to be employed in health promotion. A systematic review also found humour to be a useful tool for consideration in health promotion, particularly for conditions which are stigmatised, such as UI, but the authors also advise further research is needed due to the wide array of ways humour can be evoked [99].

As suggested in Miller et al. [99], the strength of humour lies in its apparent ability to challenge stigma and engage those experiencing conditions which have historically been hidden from public view:

*“She [the instructor] was on Woman’s Hour or something, and it was a proper lightbulb moment. She was funny, and suddenly, instead of being something shameful, it was funny, and that was so liberating”.*

The quoted participant had felt her UI was something to hide and be ashamed of, and this had had a long-term detrimental effect on her psychological and physiological well-being. However, hearing UI talked about in public and with humour had resonated with her, and as she exclaims, it was a “lightbulb moment” and “liberating”. It had only been a short time since the radio show was aired, and it appeared that the messaging broadcast was instrumental in encouraging the participant to participate in the current study.

Whilst further research is warranted, future UI interventions may want to explore the effects of using humour to reach those participants who are reluctant to engage in research on sensitive conditions.

- Need for Improved PA Infrastructure

The final theme of the study, similar to the previous one, was raised by participants unprompted and was not originally a topic considered for the current study. However, upon realising the importance to participants of having strategies in place which helped them tackle multiple barriers to their PA behaviour at once, the researcher introduced the topic to all participants. Green or active travel, for the women in the current study, was described mainly as a way to do their daily chores but also incorporate exercise to help meet their PA goals. One of the women in particular felt combining travel for chores and exercise allowed her to overcome one of the most common barriers to PA cited by women: a lack of time.

Whilst walking and cycling seemed popular with the participants, presumably because of their low intensity and likelihood of keeping leaking at bay, there were some who felt unable to benefit from this strategy and reported that their UI was a barrier to active travel:

*“A lot of things I love to do, like riding bikes and things like that, but I won’t even attempt to ride a bike at the minute”.*

This participant lived next to a national cycleway and was unable to take up the opportunity it presented for non-motorised travel due to there being “no toilets on the route”. It is unknown how long the study participants’ journeys were, but the multi-functional trips the participants described for household chores and care responsibilities are also described in the active travel literature. A UK study reported that women connected journeys between home and work but also reported women discussing undertaking other tasks en route, such as taking children to/from school or shopping, whereas men generally completed the journey between home and work directly [100]. For many women, the lack of toilet provision on many of these routes, plus the time taken to do the described additional tasks, makes active travel an impossible choice, which results in women with UI feeling like failures:

*“You try to keep some sort of level of fitness. But what upsets me the most now is that I can’t even walk”.*

The lack of toilet provision in this participant’s neighbourhood had a significant impact on her ability to undertake any PA at all. She was the only participant out of the 14 women who were interviewed who used only walking to illustrate her current PA ability; however, she was determined to do whatever she could to help her health. Listening to her talk about toilet provision in the UK, it was clear she felt let down by the lack of consideration by town and travel planners and their failure to consider the needs of women like her when planning active and public travel routes. Unfortunately, more and more women are likely going to be feeling similar frustrations at the reduction in active travel opportunities due to the current trend for closing town and city public toilets [101].

## 4. Conclusions

This study provides an understanding of the barriers to PA participation among women with UI and addresses notable gaps in the existing literature. The findings reveal a complicated relationship between physical, emotional, and social factors that contribute to PA withdrawal or modification, underscoring the challenges women with UI face in attempting to manage the condition in the PA environment. Although UI is a well-documented condition, it remains under-recognised and under-addressed in public health discourse, with particularly insufficient attention paid to the direct impact UI has on women’s reluctance to disclose their UI status. The study also identifies practitioners as having insufficient awareness of urinary incontinent women’s support needs in the PA environment which helps perpetuate a cycle of reduced PA engagement and negatively affected women’s psychological and physiological outcomes.

Survey findings revealed that only 3.2% of participants spontaneously identified UI as a barrier when asked to list all barriers affecting their PA participation. However, when prompted about UI, 38.6% of participants identified UI as a barrier, indicating a clear reluctance for women to disclose UI unprompted. In addition, over 30% of participants ranked UI within their top five barriers to PA, and almost 10% placed UI as their most bothersome or second most bothersome barrier, suggesting that whilst UI is a critical factor in influencing women’s PA behaviours, it remains underreported and underprioritised due, in part, to the pervasiveness of societal stigma and normalisation of the condition, creating fear and embarrassment which stops women from openly discussing the impact of UI on their lives.

Interview participants reported feelings of frustration, anxiety, and low self-esteem which were often exacerbated by the unpredictability of leaking urine during exercise. These emotions were a key source of anxiety, leading many women to alter their exercise behaviours in a variety of ways, such as avoiding high-impact activities or choosing low intensity alternatives such as walking. However, even low-impact activities were not free of the challenges posed by UI; the lack of accessible toilet facilities and the unpredictability of symptoms frequently disrupted participation. A recurring theme in the interviews was the inadequacy of exercise environments and exercise delivery to meet the needs of women with UI. Participants highlighted a lack of appropriate exercise modifications and insufficient practitioner knowledge on how best to support women in achieving their PA goals. The need for tailored exercise adaptations, inclusive facility designs, and practitioner training emerged as key areas for improvement. The study also shed light on the barriers to UI disclosure often reported in both clinical and PA settings. While many women expressed a willingness to discuss their condition if prompted by a knowledgeable and empathic practitioner, others voiced their concerns over judgment stigma, and the perceived inability of practitioners to provide meaningful solutions. These findings suggest that promoting an environment of trust and normalising discussions around pelvic floor health could encourage greater disclosure and support for women with UI.

The findings underscore the need for PA practitioners to proactively address UI as a barrier to participation. Training in PFMT and the provision of exercise modifications tailored to women with UI are essential to creating a more inclusive exercise environment. Practitioners should be equipped with the skills to recognise and support women with UI, creating an environment of trust and empathy that encourages disclosure. This may involve integrating questions about pelvic floor health into standard health and fitness assessments, using non-confrontational methods such as anonymous questionnaires or checkboxes to gather sensitive information. By demonstrating a thorough understanding of the condition and its implications, practitioners can build rapport with participants and reduce the stigma associated with UI. Additionally, practitioners should be encouraged to offer tailored exercise programmes that account for the physical and emotional challenges posed by UI, such as low-impact alternatives and modified group classes that minimise the risk of leaking.

At the policy level there is a pressing need to address UI as a public health priority. Campaigns aimed at destigmatising UI and raising awareness of its treatability could play a critical role in encouraging women to seek help and engage in PA. Public health messaging should emphasise the benefits of PA in managing and preventing UI, particularly through interventions such as PFMT, which can be delivered in both clinical and fitness settings.

Urban planning and infrastructure development should also consider the needs of women with UI. Ensuring adequate toilet provision and accessible facilities in PA venues and public spaces is crucial to supporting women’s participation in PA. Additionally, the design of active travel infrastructure, such as cycle paths and pedestrian-friendly routes, should incorporate toilet facilities to accommodate women with UI, particularly considering the growing emphasis on active transport and green spaces.

The current study highlights several areas for future research to further explore the relationship between UI and PA participation. Longitudinal studies are needed to examine the long-term effects of UI on women’s health behaviours and outcomes, as well as to evaluate the effectiveness of targeted interventions in reducing the impact of UI on PA participation. Qualitative research exploring the perspectives of PA practitioners and policymakers could provide valuable understanding of UI and how it impacts women’s PA, as well as aid the implementation of UI focused intervention strategies and contribute to informing the development of best practice. Further research should also investigate the experiences of diverse populations, including women from different cultural and socioeconomic backgrounds and ages, to ensure interventions are fully inclusive and address the unique challenges faced by marginalised groups. Finally, studies examining the effectiveness of different disclosure methods, such as anonymous questionnaires and verbal discussions, could help inform strategies to improve UI screening and support in PA settings.

This study possesses several notable strengths that enhance its contribution to understanding the barriers women face in PA participation due to UI. Firstly, the use of a mixed-methods design, comprising both quantitative and qualitative approaches, allowed for a comprehensive exploration of the research aims. The explanatory sequential methodology facilitated a detailed understanding of the phenomena, enabling the qualitative phase to frame and enrich the findings of the quantitative phase. This dual approach ensures the findings are both robust and reflective of the lived experiences of women with UI.

Secondly, the study’s sample size for the quantitative survey was substantial, with 345 participants recruited, ensuring a diverse and representative cohort. The recruitment strategy also drew participants from a closed social media group, capturing a varied demographic spanning different ages, backgrounds, and life stages. This diversity enhanced the transferability of findings by reflecting the broad spectrum of challenges women face in PA settings. The qualitative phase of the research further adds to the study’s depth, with semi-structured interviews providing rich, in-depth insights into women’s perceptions and experiences of UI in the PA context. The thematic analysis was guided by established frameworks ensuring methodological rigour and transparency, and additionally, efforts were made to include the few participants who spontaneously disclosed UI during the quantitative phase, strengthening the alignment of the qualitative data with the research aims.

Another strength lies in the study’s practical implications; by addressing a significant yet under-researched barrier to PA, the research provides actionable recommendations for practitioners, policymakers, and researchers. The findings emphasise the need for targeted interventions, practitioner training, and inclusive facility design, offering clear pathways to address the challenges faced by women with UI, and finally, the study contributes to reducing urinary incontinence stigma by normalising UIs discussion in PA settings. This focus on creating an inclusive and supportive environment represents an important step towards tackling sex-based disparities in health and PA participation.

Despite these strengths, the study is not without limitations. Firstly, a key limitation is related to the recruitment strategy. Whilst the use of social media enabled access to a diverse population, it also introduces potential selection bias. Participants who engage with social media groups related to health and fitness, such as the armed forces group used, may already be more motivated to discuss or address barriers to PA than the general population. Secondly, women with more severe UI symptoms or those experiencing greater stigma might also have been underrepresented in the study, as they may be less likely to engage in research involving sensitive topics. Also, the self-reported nature of the survey and interviews is a limitation, as it may have introduced recall bias or social desirability bias where participants may under-report or over-report their UI experiences or the extent to which it impacts their PA participation due to embarrassment or a desire to meet perceived social norms. Furthermore, while the study highlights the importance of practitioner training and awareness, it does not directly include the perspectives of PA practitioners. Understanding practitioner knowledge, attitudes, and barriers to addressing UI in PA settings would have added valuable insights to complement the women’s perspectives and should be considered for future exploration, and finally, the study design does not include longitudinal follow-up. The cross-sectional nature of the data limits the ability to assess changes in behaviour or perceptions over time, particularly in response to targeted interventions or shifting cultural attitudes towards UI.

In conclusion, this study provides a comprehensive understanding of the barriers posed by UI to women’s PA participation and highlights the significant physical, emotional, and social challenges faced by this population. The findings emphasise the urgent need for practitioners, policymakers, and researchers to prioritise UI as an essential component of PA intervention design and delivery. By addressing the stigma and misunderstandings surrounding UI, nurturing inclusive and supportive environments, and equipping practitioners with the necessary skills and knowledge, it is possible to empower women to maintain active lifestyles and achieve their health and well-being goals. Fundamentally, addressing UI as a barrier to PA requires a collaborative approach that integrates public health, clinical, and fitness perspectives. By implementing targeted interventions and policies, we can improve PA participation among women with UI and contribute to reducing sex-based disparities in health outcomes by promoting a more equitable and inclusive approach to PA and health.

## Figures and Tables

**Table 1:** Survey participant demographic characteristics.

	Frequency	% of N	Mean	Min	Max
Age (years old) (n = 345)			47.7	18	79
18–35	21	6.1			
36–55	272	78.8			
56–65	32	9.3			
>65	20	5.8			
Education Level (n = 345)					
Secondary Education or less	61	17.7			
Further Education	17	4.9			
Undergraduate	182	52.8			
Postgraduate	85	24.6			
Self-Reported Physical Activity (minutes/week) (n = 189) *			179.21	0	1440
No Exercise	23	12.2			
1–149	87	46.0			
150–300	48	25.4			
301–600	26	13.8			
>600	5	2.7			

* Subjectively reported PA levels include any activity where breathing was perceived to be laboured, including housework and walking.

**Table 2:** Interview participant demographic characteristics (n = 14).

	Frequency	% of N	Mean	Min	Max
Age (years old)			55.29	46	68
46–55	8	57.1			
56–65	3	21.4			
>65	3	21.4			
Education Level					
Secondary Education or less	2	14.3			
Further Education	8	57.1			
Undergraduate	2	14.3			
Postgraduate	2	14.3			
Self-Reported Physical Activity (minutes/week) *			176.43	0	420
0–59	2	14.3			
60–149	5	35.7			
150–300	4	28.6			
301–600	3	21.4			
Leak Urine During Physical Activity					
Yes	14	100			
No	0	0			

* Subjectively reported PA levels include any activity where breathing was perceived to be laboured, including housework and walking.

## Data Availability

The data presented in this article are not readily available due to privacy restrictions. Requests to access the datasets can be directed to the first author.

## Abbreviations

The following abbreviations are used in this manuscript:

PA	Physical Activity
UI	Urinary Incontinence
NCD	Non-communicable disease
WHO	World Health Organisation
PAH	Physical Activity and Health
NHS	National Health Service
QOL	Quality of Life
